# Even Warriors Can be Scared: A Survey Assessing Anxiety and Coping Skills in Competitive CrossFit Athletes

**DOI:** 10.3390/ijerph17061874

**Published:** 2020-03-13

**Authors:** Jan Wilke, Tatjana Pfarr, Mandy-Deborah Möller

**Affiliations:** Department of Sports Medicine, Goethe University Frankfurt, 60487 Frankfurt am Main, Germany; tatjanapfarr@gmail.com (T.P.); moeller@sport.uni-frankfurt.de (M.-D.M.)

**Keywords:** cross fitness, psychology, competition fear, motivation

## Abstract

Competition anxiety has been demonstrated to decrease sports performance while increasing burnout risk. To date, its degree in CrossFit (CF) is unknown. The present study, therefore, examines competition fear and relevant coping skills as well as potential correlates of both in individuals participating in CF events. A total of *n* = 79 athletes answered a battery of three questionnaires (competition fear index, athletic coping skills inventory, mindfulness attention awareness scale). Substantial levels of anxiety, particularly regarding the somatic dimension of the competition fear index, were reported. The most pronounced coping skill was freedom of worry. While age or level of competition showed no/very small associations with survey data, sex was correlated to the psychological characteristics: women reported higher competition fears and lower coping skill levels (*p* > 0.05). Competition fears are highly prevalent in CF athletes and the preventive value of population-specific interventions, particularly in females, should be investigated in future trials.

## 1. Introduction

CrossFit (CF) is a highly popular conditioning program combining elements of strength, coordination, balance and mobility [1,2]. It represents one of the most common examples of high-intensity interval training, which has recently been ranked second in surveys of both worldwide and European exercise trends [3,4]. According to focused analyses, CF grows faster than the world’s largest fitness franchise [1]. In addition to bodily adaptations, recent research has also started to examine psychological aspects of CF performance [5,6,7]. 

Besides improving health and fitness, competition represents a key motivation for a substantial share of CF athletes [7]. Interestingly, in a variety of sports (running, skiing, track and field, swimming, basketball, tennis, football), considerable levels of competition anxiety have been demonstrated [8,9,10]. Among the different types, the magnitude of fear is higher in individual when compared to team sports [11]. While arguably creating situational discomfort, anxiety can have even more severe consequences than this, causing performance decrements [8,9,10] and a higher risk for burnout [12,13]. The development of abilities aiming to counteract or reduce competition fear is thus highly relevant from both a success-oriented and a health-centered perspective. In fact, a plethora of coping skills (e.g., freedom of worry, concentration, mindfulness) are negatively coupled with anxiety, meaning that their development could help affected athletes [14,15]. 

Several coping skills may be of relevance for CF competitors. Concentration, freedom of worry and coachability have been demonstrated to discriminate successful and unsuccessful athletes in football [16]. Concentration represents the ability to focus on a specific moment, freedom of worry describes the capacity to suppress negative thoughts and coachability is characterized as being open for learning, reflecting own actions and using external feedback [16]. As CF competitions include a variety of constantly changing tasks which are to be performed in the presence of spectators and judges, all three may be of particular relevance for the athletes. In addition to the named above, other skills may also help CF competitors in counteracting anxiety. For instance, mindfulness is a state of attentiveness and receptiveness towards what is happening around oneself [15]. It has been shown to be beneficial in sports requiring precision, which is central to the correct execution of loaded movement patterns as occurring in CF. The effectiveness of mindfulness training to increase sports performance has been demonstrated in a systematic review with meta-analysis [17]. 

Both the degree of competition anxiety and the strength of related coping skills seem to be modified by contextual (e.g., performance level) and intrinsic (e.g., sex) variables [18,19,20,21,22,23]. For anxiety, among the contextual factors, greater training volumes and higher performance levels appear to be predictive [18]. When considering intrinsic factors, sex represents an interesting variable with ambiguous evidence: Whilst some studies point towards a higher prevalence in men [18], others reported stronger anxiety in women [19,20,21], or no difference at all [23]. With regard to coping, it has been shown that a higher level of the contextual factor performance is associated with better skills. Similar observations have been made for the intrinsic factor age: older athletes outperform younger individuals when dealing with competition fears [24]. 

In contrast to other sports, the presence of competition fear and the strength of coping abilities is scarcely described in CF. Gaining insight into these factors would be valuable in order to increase sports-related performance and prevent the possible development of psychological symptoms such as burnout. The present study, therefore, had three objectives. In a first step, we aimed to describe the level of anxiety in CF performers engaging in competition. The second issue consisted in identifying weakly or strongly developed coping skills, potentially counteracting these fears. Finally, the study was undertaken to reveal the influence of age, sex and performance level on both levels of fear and coping skills. 

## 2. Materials and Methods 

### 2.1. Participants

A total of *n* = 79 athletes (32.7 ± 7.3 years, 43 females) were enrolled. Recruitment was performed in four German CF facilities using flyer advertising and, additionally, personal addressing. Regarding the latter, to prevent a selection bias, all members of the participating facilities were approached by word of mouth. Inclusion criteria were (1) age of 18 years or older and (2) at least one participation in an organized CF competition during the last 12 months.

In summary, the included athletes were highly active, reporting 4.9 ± 1.2 training sessions with a total duration of 8.9 ± 4.2 h per week. On average, they participated in 2.9 ± 1.9 competitions during the last year. The largest part of the sample were high-level athletes (elite, 50.6%), followed by low- (scaled, 25.3%) and mid-level athletes (masters/masters+, 24.1%). Slightly more than half of the participants (53.2%) stated regular engagement in other sports, with running (*n* = 12) and cycling (*n* = 6) being most popular. Performance-related details are presented in Table 1. 

### 2.2. Procedures

A paper-based survey following the recommendations for Good Practice in the Conduct and Reporting of Survey Research [26] was performed. Ethical approval was obtained from the local committee (German University for Health and Sport, reference number: 01/2018.91002800) and all participants provided written informed consent. 

Assessments took about 10 to 15 min and were performed in a quiet room. Only the study personnel were present. All participants completed three questionnaires. 

The Wettkampf–Angst index (WAI, competition-fear index) represents a measure of anxiety in sports. It captures the agreement with 14 claims regarding nervousness, excitement and doubts, potentially arising prior to a competition (4-point Likert scale). Three scores are computed, indicating somatic fear, concernedness and concentration disturbances. High reliability (α = 0.77–0.83) and significant convergence validity of the instrument have been demonstrated [27]. Cronbach’s alpha in the present study was 0.74 for concentration disturbances, 0.81 for somatic fear, 0.79 for concernedness and 0.70 for the total score. 

Coping skills were assessed with two questionnaires. The Athletic Coping Skills Inventory (ACSI) has 28 items to be answered on a 4-point Likert scale (0: almost never to 3: almost always). It produces an overall score of coping with diverse sport-related pressures and threats and additionally yields seven subscales (coping with adversity, peaking under pressure, goal setting and mental preparation, coachability, concentration, confidence and achievement motivation, freedom of worry). Both reliability (α = 0.87) as well as construct and predictive validity of the questionnaire have been documented [28]. Cronbach’s alpha in the present study was 0.64 for coping with adversity, 0.75 for coping with adversity, 0.73 for goal setting and mental preparation, 0.81 for coachability, 0.72 for concentration, 0.63 for confidence and achievement orientation, 0.71 for freedom of worry and 0.81 for the total score.

While the ACSI covers several relevant coping abilities, it does not capture mindfulness. It was therefore assessed by means of the Mindfulness Attention Awareness Scale (MAAS). The instrument consists of 15 items, which are to be answered on a 6-point Likert scale (1: almost always to 6: never). The German version has high internal consistency (α = 0.83) and construct validity [29]. In the present study, Cronbach’s alpha was 0.89.

### 2.3. Statistical Analysis

Descriptive and inferential statistics were used to analyze the obtained data, as appropriate. Interval/quasi-interval scaled data (e.g., age, pain intensity) and dichotomous data (e.g., presence of pain) were reported as absolute (n) and relative (%) values, respectively. Survey results were descriptively compared against the mean values of athletic samples from norm value and validation studies [27,28,29]), see Table 2. Differences between males and females in the three scores were examined using the Mann–Whitney U test. In case of statistical significance, effects’ sizes were calculated by means of the formula *r* = z/√n. Resulting values were interpreted as small (0.1), medium (0.3) or large (0.5), according to Rosenthal [30]. To detect systematic associations of the assessed variables, Spearman’s rho coefficients, including 95% confidence intervals (95%CI), were estimated. For data analysis, the software BiAS Statistics 11.10 (Goethe University, Frankfurt/Main, Germany) was used for all analyses and significant associations among study variables were inferred at α = 0.05.

## 3. Results

The participants’ scores on the five questionnaires are displayed in Table 2.

### 3.1. Competition Fear

The highest values, exceeding the mean of the norm data [28], were reported for the somatic sub scale of the WAI, followed by concentration disturbance (exceeding the norm data) and concernedness (almost identical to norm data). Compared to men, women reported higher somatic fear (median: 13 vs. 10, *p* = 0.001, *r* = 0.38) and concernedness (11 vs. 8, *p* = 0.01, *r* = 0.29), but there was no difference regarding concentration disturbance (*p* = 0.90). Training volume was negatively associated with concernedness (*p* = 0.03, rho = −0.24, 95%CI: −0.02 to −0.47) and concentration disturbance (*p* = 0.01, rho = −0.28, 95%CI: −0.06 to −0.50). In contrast to the other two dimensions, higher performance levels were interrelated with less concernedness (*p* = 0.03, rho = 0.25, 95%CI: 0.02 to 0.48). No associations for competition fear and age were found (*p* > 0.05). 

### 3.2. Coping Skills

When compared to the norm data [29], analysis of the ACSI revealed above-average values for freedom of worry and below-average values for confidence and achievement orientation, while the other coping skills corresponded to the average. Women reported lower coping skills (median: 45.5 vs. 54) than men (*p* = 0.016, *r* = 0.28). The same observation was made in the subscales’ concentration (6 vs. 9, *p* = 0.04, *r* = 0.23), confidence (6 vs. 7, *p* = 0.04, *r* = 0.23) and goal setting (5 vs. 7, *p* = 0.02, *r* = 0.27). 

Higher training volumes correlated with higher coping skill values (*p* = 0.004, rho = 0.33, 95%CI: 0.11 to 0.54), particularly confidence (*p* < 0.001, rho = 0.51, 95%CI: 0.32 to 0.71), goal setting (*p* = 0.001, rho = 0.38, 95%CI: 0.17 to 0.59) and speaking under pressure (*p* < 0.001, rho = 0.42, 95%CI: 0.21 to 0.63). No association of age and coping skills was detected, neither for the total score (*p* = 0.55), nor for the subscales (*p* > 0.05). Similarly, performance level was not related to coping skills, neither the total score nor the subdomains (*p* > 0.05), except for peaking under pressure, which was associated with higher-level performance (*p* = 0.04, *r* = 0.24). 

The mean score on the MAAS scale revealed mindfulness values similar to those of norm populations [27]. Slightly higher mindfulness was found for women (median: 65.0 vs. 61.5 points) when compared to men (*p* = 0.049, *r* = 0.23). Neither training volume (*p* = 0.94) nor age (*p* = 0.84) or performance level (*p* = 0.87) were associated with mindfulness (*p* = 0.84). 

## 4. Discussion

To the best of our knowledge, there have not been systematic assessments of anxiety in CF athletes, so far. The main finding of the present study is that competitive athletes seem to display substantial contest-related fears. 

Previous investigations into the occurrence of anxiety revealed the highest levels in individual sports [11]. Although the reasons are still a matter of debate, it could be argued that here, the focus of the audience is not distributed between multiple athletes and that own failures cannot be compensated by teammates [11]. This (a) particularly applies to CF, as performance is centered around improving personal records and (b) may explain the high anxiety values found in our sample. When considering the sub-dimensions of competition fear, the greatest values were registered for the somatic aspect, which is characterized by bodily reactions such as palpitations, perspiring hands or an upset stomach. In addition to this, above-average levels of fear were also found for concernedness, expressing the tendency to develop self-doubts or negative expectations. In contrast, ratings of concentration disturbances almost corresponded to the average. Although this hypothesis should be tested in follow-up investigations, the observed pattern (somatic manifestations and self-doubts/negative expectations but no concentration impairments) may indeed be triggered by social pressures. 

Besides the generally considerable levels of competition fear, another result of our analysis is that women may be more at risk for high anxiety and low coping than men. While other variables, such as age, training volume and performance level had only a minor impact on most measured outcomes, there were marked differences between the sexes: female athletes reported stronger fears. Particularly the somatic aspect seems to represent a significant issue in women as they, with 13 out of 16 points, reached a score close to the maximum. When compared to men, women have been shown to underestimate their performance capacity in muscular tasks [31]. Such rather pessimistic evaluation of their own potential could increase the self-perceived difficulty of the competition and thereby, explain the difference in anxiety levels. With regards to published literature, Russel et al. [19] found mixed results overall, but higher somatic fears in women prior to play-off ball games when compared to men, which would fit with our data. Additionally, Silva [20] found slightly higher values for fear of success in female undergraduate athletes when compared to their male counterparts, and Kristjánsdóttir et al. [21] measured lower anxiety levels in male elite handball players. In contrast, neither Sagar et al. [22] in British university student athletes nor Smith [23] in junior team sport athletes found differences as a function of sex. 

When compared to normative data [29], the coping skills of the investigated sample could be classified as mostly corresponding to the average. However, lower values were reported for confidence and achievement orientation. As learning and mastering skills represents an important hallmark of the motivational profile in CF [7], and as specific skills are to be demonstrated in competition, the lack of confidence and achievement orientation could explain the increased anxiety levels. When considering the role of sex, the high fears of women are of particular relevance because females also stated lower coping skills than men. Results of studies examining coping differences between males and females are similarly ambiguous as those regarding the level of anxiety. A systematic review elucidating sex differences in sports-related coping skills concluded that there is no undisputable proof of a different behavior between men and women [24]. In any case, the sex differences found in the present study warrant further investigation. 

The findings of our study have implications for clinical practice. When analyzing the loads acting on CF athletes, it needs to be emphasized that these are by no means exclusively physical. CF performers may consider implementing adjunctive psychological interventions such as mindfulness or anti-stress training which could help to (a) increase performance [32] and (b) prevent the development of chronic stress syndromes. The latter is important because it has been shown that competitive state anxiety may predict the occurrence of some aspects of burnout [13]. Besides the athletes, our results may also represent a call for action for the owners of CF studios and coaches working in related facilities. Support and counseling for athletes preparing for competitions should address an individual assessment of the potentially performance-hampering threats and fears, irrespective of the performance level.

Some limitations of the present study have to be acknowledged. We compared our survey results against published norm data [27,28,29]. However, as there is a lack of specific cut-offs identifying potentially ‘pathological’ value ranges, it needs to be underlined that our investigation allows a descriptive comparison against the norm, but is still qualitative in nature. With 75 participants, the sample size was small and the inferences drawn from our findings need to be interpreted with caution when aiming to generalize them to other CF performers. It would hence be intriguing to conduct further studies with larger participant numbers in order to confirm the results. Another issue is that we did not randomize the order of questionnaire administration. Although the total time to complete all scales was not very long, this may have led to a bias, affecting the response behavior in the final phase of the assessments [33]. Finally, all data were collected based on self-report of the participants. Future studies may apply a triangulation-based approach integrating assessments of other persons, i.e., psychologists.

## 5. Conclusions

Competition anxiety is a frequent finding in CF athletes. While factors such as age and performance level do not seem to significantly impact the nature and magnitude of the self-reported fears, women may be more vulnerable than men. In view of the sport’s high popularity, future research should elucidate the impact of anxiety on injury risk and performance, as well as the value of interventions specifically tailored to reduce its symptoms.

## Figures and Tables

**Table 1:** One-repetition maximum of the survey participants for different CrossFit exercises as a function of competition level.

Exercise	Elite	Masters	Masters +	Scaled	Total
Clean and Jerk	92 ± 30.6	85.4 ± 20.9	86 ± 33.3	56.5 ± 26.2	81.8 ± 31.3
Snatch	68.8 ± 27.6	64.8 ± 17.5	64.5 ± 24.7	43.9 ± 16.1	61.5 ± 25.0
Back Squat	122.6 ± 42.8	118.1 ± 31.4	113.3 ± 37.9	85.0 ± 40.2	111.8 ± 42.3
Deadlift	160.9 ± 42.5	155.5 ± 41.2	146.7 ± 58.0	110.0 ± 41.6	146.4 ± 47.0
Strict Shoulder Press	59.3 ± 19.8	58.2 ± 15.1	60.8 ± 27.4	38.6 ± 21.7	53.9 ± 21.3

All values are reported in kilograms. Masters +: 45 years and older. Performance classification based on CrossFit world cup [25].

**Table 2:** Descriptive statistics of the survey result.

Questionnaires and Related Sub-Scales	Scale Range	Mean	SD	Minimum	Maximum	Reference Data
**WAI**						
Somatic fear	4 to 16	11.3	2.9	5	16	M: 7.9, F: 9.1
Concernedness	4 to 16	9.4	3.3	4	16	M: 7.6, F: 7.9
Concentration disturbances	4 to 16	6.2	1.9	4	11	M: 6.4, F: 6.1
**MAAS**						
Total score	15 to 90	63.3	13.0	37	90	M/F: 64.3
**ACSI**						
Coping with adversity	0 to 12	6.4	2.7	0	12	M/F: 6.2
Coachability	0 to 12	9.4	2.4	4	12	M/F: 9.3
Concentration	0 to 12	7.3	2.2	3	12	M/F: 7.0
Confidence and achievement motivation	0 to 12	6.9	2.3	0	12	M/F: 7.9
Goal setting and mental preparation	0 to 12	5.9	3.1	0	12	M/F: 5.6
Peaking under pressure	0 to 12	6.9	2.7	0	12	M/F: 6.5
Freedom of worry	0 to 12	7.5	2.9	0	12	M/F: 5.8
Total score	0 to 84	50.3	12.7	80	15	M/F: 48.3

WAI: Wettkampf–Angst index (Competition-Fear-Index), MAAS: mindfulness attention awareness scale, ACSI: athletic coping skills inventory. M = males, F = females. Reference data column shows mean values from validation studies [25,26,27].
